# Assessment of Human Taeniasis and Other Intestinal Parasites in Narok County, Kenya

**DOI:** 10.1155/vmi/9226601

**Published:** 2025-03-29

**Authors:** D. O. Oduori, P. M. Kitala, T. M. Wachira, E. Mulinge, T. Irungu, E. Zeyhle, R. Ofwete, S. Gabriël, P. B. Gathura

**Affiliations:** ^1^Department of Public Health Pharmacology and Toxicology, University of Nairobi, Nairobi, Kenya; ^2^Department of Animal Health and Production, Maasai Mara University, Narok, Kenya; ^3^Kenya Medical Research Institute, Nairobi, Kenya; ^4^Department of Public Health, Meru University of Science and Technology, Meru, Kenya; ^5^Amref Health Africa, Nairobi, Kenya; ^6^Department of Translational Physiology, Infectiology and Public Health, University of Ghent, Merelbeke, Belgium

**Keywords:** intestinal parasites, Kenya, taeniasis

## Abstract

Data are sparse on the epidemiological picture of *Taenia saginata* taeniasis in Kenya. Infections are underreported, and their persistence nonetheless negatively impacts the beef industry. Populations vulnerable to taeniasis in the developing world are commonly burdened with other intestinal parasites, ubiquitous in unsanitary environments. This study aimed to estimate the occurrence of human taeniasis in Narok County, Kenya, and screen for the presence of other intestinal parasitic infections. A community-based survey was conducted in five pastoral wards, and stool samples, mainly from adults, subjected to multiple diagnostic tests. One sample tested positive for *Taenia* spp. by coproantigen enzyme-linked immunosorbent assay (0.3%, 95% CI, 0–1.6, *n* = 360), and all samples tested negative on multiplex copro-polymerase chain reaction targeting the cytochrome c oxidase subunit 1 gene and copromicroscopy. Microscopy (*n* = 361) additionally identified *Entamoeba histolytica*/*dispar*/*moshkovskii* at a prevalence of 15.5% (95% CI, 12.1–19.6), *Giardia* spp. at 5.3% (95% CI, 3.4–8.1), *Hymenolepis* spp. at 1.1% (95% CI, 0.4–2.8), and hookworm at 0.3% (95% CI, 0–1.6). Grazing livestock near the homestead (< 2 km) and a formal education background were associated with a reduced likelihood of *Giardia* spp. infection (AOR 0.07, 95% CI 0–0.36, *p*=0.011, and AOR 0.06, 95% CI 0.01–0.50, *p*=0.014, respectively). Our findings suggest a very low prevalence of human taeniasis in the population. The occurrence of other pathogenic zoonotic intestinal parasites highlights a public health concern and calls for a One Health approach in the enhancement of hygiene initiatives.

## 1. Introduction

*Taenia saginata* taeniasis is an ancient cyclozoonoses, with paleoparasitological records dating infection to as early as 3200 years ago, coinciding with the domestication of cattle from its ancient ancestor, the African aurochs (*Bos primigenius*) [1, 2]. The helminth infection is persistent and currently has a global distribution, estimated to affect at least 50 million people worldwide with infection foci in Africa, the Middle East, and parts of Europe [3]. Its health impact is however relatively low [4, 5]. Humans acquire infection by ingesting raw or undercooked beef containing viable larvae, with expulsion of gravid proglottids in stool and/or their migration to the anus after 3–4 months [6, 7]. In cattle, infection is associated with significant postharvest losses stemming from carcass condemnations and restrictions on exports [8].

The occurrence of human taeniasis in Kenya has displayed varying trends over time and across different regions. Nevertheless, there is a scarcity of surveillance data available. Previous surveys have primarily relied on microscopy for detection and subsequent estimation of prevalence. Estimates have ranged from 0% to 68%, from 1965 to 2016, and the highest estimate (68%) was reported from older studies. More recent studies (2006–2016) have reported relatively lower estimates, that is, 0%–19.7% [9–19]. Few studies have focused on taeniasis in the human population of Narok County, despite the region being characterized as having “hyperendemic pastoral cysticercosis.” An epidemiological scenario denoted by a simultaneous high burden of *T. saginata* infection in humans and cattle [3, 9]. Old reports by Froyd [9] had estimated the prevalence of taeniasis due to *T. saginata* at 28% and bovine cysticercosis at 53% in Narok region. Rates as high as 31% have also been reported in the area for bovine cysticercosis [20]. Narok County has one of the highest densities of cattle in Kenya, representing approximately 9% of the country's total cattle population [21] and therefore a significant contributor to the country's food security.

Few studies have employed immunodiagnostics for epidemiological surveillance of taenia in the country. Tembo [13] reported a *Taenia* spp. prevalence of 5.9% using coproantigen ELISA (coproAg-ELISA) in a study conducted in 2007. Subsequent studies conducted in western Kenya in 2010 revealed prevalence estimates of 19.7% and 1.9% [16, 19]. The application of copromicroscopy and coproAg-ELISA as the primary detection tools in the above studies leaves the true prevalence of *T. saginata* taeniasis in contention as the tests are only genus-specific. Whereas copromicroscopy relies on the morphological identification of *Taenia* spp. eggs in stool, coproAg-ELISA responds to *Taenia* spp. antigens and is therefore capable of detecting prepatent cestodes in the gut [22, 23]; both tests, however, cannot differentiate between *T. saginata* and *Taenia solium* infections. Yamasaki et al. [24] have documented the use of molecular tools capable of detecting *Taenia* spp. DNA in stool samples. However, studies utilizing such molecular techniques for surveillance purposes in Kenya remain scarce [12, 19].

Resource-poor populations, often characterized by inadequate sanitation infrastructure, low health literacy levels, and close proximity to livestock or wild animals, are particularly vulnerable to multiple intestinal parasitic pathogens that share transmission pathways [25]. Among the most common protozoan intestinal parasites affecting these populations are *Giardia lamblia* and *Entamoeba histolytica.* Additionally, soil-transmitted helminths (STH) remain prevalent in such environments [26–28].

*Giardia lamblia*, also known as *Giardia intestinalis* or *Giardia duodenalis*, is a cosmopolitan protozoan parasite that infects both humans and animals. It exists as a flagellated diplomonad trophozoite and as a cyst. The cyst form colonizes the small intestine upon ingestion. In humans, infection can be asymptomatic or manifest as chronic diarrhea and malabsorption [29]. Infection has been associated with 171,000 Disability-Adjusted Life Years (DALYs) [26–28, 30]. In Kenya, the occurrence of giardiasis ranges between 4% and 39% with the heaviest infection burden identified in western Kenya [30–37]. The most vulnerable groups associated with infection have been children and people living in rural or informal settlements [30]. Anthropozoonotic spread has been suggested in Kenya, with infections in humans linked to contact with domestic cattle, goats, and poultry [38].

*Entamoeba histolytica* is another common intestinal protozoal parasite. Like *G. lamblia*, humans acquire infection via the fecal–oral route. In the intestinal lumen, the parasite excysts into its mobile, pathogenic trophozoite form. While most infections are asymptomatic, untreated *E. histolytica* infections can lead to fatal extraintestinal amebiasis [39]. This parasite is responsible for a disease burden of 516,000 DALYs [27, 28]. In Kenya, infection rates range from 9% to 36%, with studies primarily focusing on children under 18 years living in rural and informal settlements, who are particularly vulnerable to infection [31, 32, 34–36, 40].

STH (*Ascaris lumbricoides*, *Trichuris trichiura,* and hookworm species) similarly persist in unsanitary environments. *Ascaris lumbricoides* and *T. trichiura* are transmitted via the fecal–oral route, whereas infective hookworm larvae (*Ancylostoma duodenale* and *Necator americanus*) penetrate the skin [41]. The global burden of disease attributed to STH is 5.18 million DALYs, with children being disproportionately affected [26]. Infection may lead to chronic malnutrition, stunting, decreased physical fitness, and reduced cognitive development [41]. In Kenya, STH are endemic in 66 subcounties [42, 43]. Narok County has reported a slow decline in STH prevalence following mass drug administration (MDA) initiatives. Surveillance in school children showed a prevalence of 34.3% in 2019 after seven rounds of MDA from 2012 to 2018, down from a baseline prevalence of 53% in 2012 [43, 44]. Our review did not identify published reports on the occurrence of intestinal protozoal infections in the human population of Narok County nor on the occurrence of STH in adults from the region.

This study aims to enhance the existing knowledge on the epidemiology of *T. saginata* taeniasis in at-risk cattle-owning communities of Narok County. To increase the sensitivity and specificity of detection, multiple methods, including molecular tools, were utilized. Additionally, screening for other intestinal parasites of public health importance was conducted.

## 2. Materials and Methods

### 2.1. Study Area

**Figure 1:**
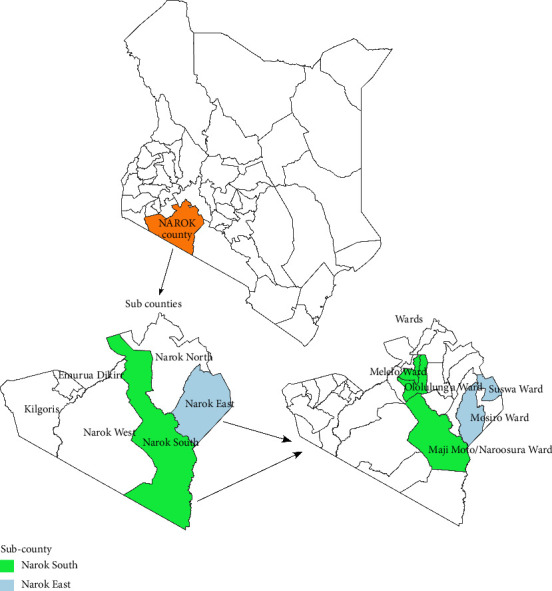
Map of Kenya depicting Narok County and wards where sampling was conducted.

The study was conducted in Narok County, Kenya, located at coordinates 1°10′S 35°37′E, with a total area of 17,921.2 square kilometers. The county hosts approximately 1,157,873 people, with only 35% having access to improved sanitation and 20% to improved drinking water sources. Narok County comprises six subcounties: Kilgoris, Narok North, Narok South, Narok East, Narok West, and Emurua Dikirr (see Figure 1). The region experiences an ambient temperature range of 12°C–28°C and an annual rainfall average of 500–1800 mm. Economic activities include mining, crop farming, tourism, and livestock farming, with pastoralism being a widely practiced production system [21].

### 2.2. Sampling and Sample Size Determination

A cross-sectional study design was employed. A sample size of 384 was utilized as per the computation output of the formulae by Thrusfield [45] where the expected prevalence (Pexp) was set at 50% to maximize the sample size, the desired absolute precision (d) set at 0.05, and the confidence level at 95% to give a sample size of 384 individuals. Study participants were required to provide a stool sample. Contextual information on the study was collected through the use of questionnaires. Some of the variables documented included cattle grazing distance from home, grazing system, water source for livestock, gut symptoms experienced before seeking treatment, distance of water source from homestead, latrine presence or absence, meat preparation method, age, position in the household, and education level.

Study participants were selected from pastoral communities residing in Narok East and Narok South subcounties. A multistage approach was used to allocate study participants. Within these subcounties, 50% of their wards were selected at random that is, Suswa and Mosiro Wards for the Narok East subcounty and Ololulung'a, Melelo, and Maji Moto–Naroosura Wards for Narok South subcounty. The sublocations selected in Narok East were Suswa, Ntulele, Kilonkisa, Enkoireroi, and Ongata Nando and the sublocations selected within Narok South were Nkoben, Melelo, Enkiu, Nkimpa, Ilkerine, Enturoto, and Oloirouwa. Subsequently, villages within these sublocations were selected at random. Households within the villages were selected through systematic random sampling, and the number of households to be sampled per village was proportionally allocated. The household head was preferably sampled per household.

### 2.3. Sample Collection

Sampling was conducted from June to July 2022. Each study participant was issued with two labeled receptacles, one containing 10% formalin, diluted in phosphate-buffered saline (PBS) pH 7.2 containing 0.3% Tween 20, and the other with 80% ethanol for the collection of stool samples. These were retrieved a day later. All samples were initially gathered at designated medical health facilities and then transported to KEMRI in Nairobi, where they were stored at room temperature until processing.

### 2.4. Coproscopic Examination

An adaptation of the formalin-ether concentration technique, as described by Truant et al. [23] and Cheesbrough [46], was employed to detect parasites in stool samples previously preserved in formalin. Briefly, each stool sample, weighing 1.0–1.5 g, was suspended in 10 mL of 10% formalin in a centrifuge tube. The suspension was then filtered through two layers of prewet surgical gauze into a new centrifuge tube. Additional formalin (10%) was added to bring the volume to 7 mL, followed by the addition of 3 mL of diethyl ether. After mixing, the suspension was centrifuged for 10 min at 785 g. This centrifugation resulted in a four-layered suspension, from which the sediment stratum was retained after decantation. A drop of the sediment was then examined under a microscope at x10 objective, and when necessary, an iodine-stained suspension was introduced in the field to aid in identification. Higher magnification was utilized for the morphological detection of suspicious objects. Morphological identification was facilitated by reference to depictions provided by Cheesbrough [46] and Garcia [47].

### 2.5. DNA Extraction

All stool samples, previously collected and stored in receptacles containing 80% ethanol, underwent DNA extraction using the QIAamp Fast DNA Stool Mini Kit protocol (Qiagen, Hilden, Germany) with specified modifications. The protocol utilized 600 μL of stool supernatant, 25 μL of proteinase K, 600 μL of Buffer AL, 100 μL of ethanol (96%–100%), and 100 μL of Buffer ATE. The extracted DNA was then transferred to labeled collection tubes and stored at −20°C pending subsequent tests.

### 2.6. *Taenia* spp. Multiplex Polymerase Chain Reaction (mPCR)

A multiplex coproPCR adapted from Yamasaki et al. [24] targeting the cytochrome c oxidase subunit 1 gene was utilized. The test uses multiple primer sets within a single PCR mixture to produce amplicons of varying sizes that are specific to DNA sequences of *T. saginata* (827 bp) and *T. solium* (American/African (720 bp) and Asian genotypes (984 bp). The primers used were 5′-TTGATTCCTTCGATGGCTTTTCTTTTG-3′ (TsagF), 5′-GGTAGATTTTTTAATGTTTTCTTTA-3′(Tsol/AmerF), 5′-TTGTTATAAATTTTTGATTACTAAC-3′ (Tsol/AsiaF), and 5′-GACATAACATAATGAAAATG-3′ (TaeniaR).

The amplification mixture volume per reaction was constituted to a total of 25 μL that is, a DNA template of 5 μL mixed with a cumulative mastermix of 20 μL. The 2 × Qiagen multiplex PCR master mix (Qiagen, Hilden, Germany) was used at 12.5 μL, 10 × primer mix at 2.5 μL (1 ×), and RNase-free water at 5 μL constituted as indicated by the manufacturer. Amplification was conducted using Applied Biosystems Veriti Thermal Cycler. The settings were as follows: an initial denaturation step at 95°C for 15 min, followed by 40 cycles at 94°C for 30 s, 58°C for 90 s, and 72°C for 90 s, and a final extension at 72°C for 10 min. To visualize the amplicons, electrophoresis was conducted on impregnated 2% (w/v) agarose gels at 120 V for 60 min, followed by ethidium bromide staining for 20–30 min and photography under UV illumination.

### 2.7. Coproantigen Enzyme-Linked Immunosorbent Assay

Samples for this procedure were aliquoted from stool samples previously collected at stored in 10% formalin, diluted in PBS pH 7.2 containing 0.3% Tween 20. The procedure was adapted from the description by Allan et al. [22] and Mwape et al. [48]. Initial processing of the samples was achieved by first mixing an equal amount of PBS with stool stored in 10% formalin and allowing the mixture to soak for 60 min with intermediate shaking, then centrifuging at 2000 g for 30 min. The supernatant was then used in the ELISA. Polystyrene ELISA plates (Immunoplate Maxisorp F96) were coated with the capturing hyperimmune rabbit anti-*Taenia* IgG polyclonal antibody (PoAb Rabbit SGL-1 IgG) diluted at 2.5 μg/mL in carbonate–bicarbonate coating buffer (0.05 M, pH 9.6). The plates were then incubated for 60 min at 37°C, washed once with PBS in 0.05% Tween 20 (PBS-T20, 0.05%) (washing buffer), and all wells blocked by adding blocking buffer (PBS-T20, 0.05% + 2% Newborn Calf Serum). After incubating at 37°C for 60 min and without washing, 100 μL of the stool supernatant was added (paired), at designated wells, and the plates incubated for 60 min at 37°C. This was followed by washing five times with the washing buffer. Biotinylated hyperimmune rabbit IgG polyclonal antibody (PoAb Rabbit SGL-1 IgG-Biot) diluted at 2.5 μg/mL in blocking buffer was used as the detecting antibody at 100 μL in designated wells after which the plate was incubated for 60 min. This was followed by washing five times with washing buffer. One hundred microliters of streptavidin–horseradish peroxidase (Jackson Immunoresearch Lab, Inc.) diluted at 1/10,000 in blocking buffer was added as conjugate in designated wells. After 60 min of incubation at 37°C, followed by washing 5 times, 100 μL of ortho-phenylenediamine (OPD) substrate was added. The plates were incubated in a dark recipient for 15 min at 30°C before stopping the reaction by adding 50 μL of sulfuric acid (4 N) to all wells. The plates were read using an automated spectrophotometer at 490 nm with a reference of 655 nm. As all samples were done in duplicate, an average OD was calculated for every sample, and the cutoff was set at 0.972 OD.

### 2.8. Data Handling and Analysis

The data were entered into a Microsoft Excel spreadsheet for data cleaning before exporting into R software version 4.3.3 for analysis. Summary statistics of variables were generated as frequencies and percentages. Chi-square/Fisher's exact test for independence was used to assess for associations between the grouping variable and the presence/absence of a parasitic infection of interest. A binary logistic model was used to test for the effect of all variables on infection. Simple models were initially developed to identify crude associations and then later tested for significance in an adjusted model. A forward selection method was applied to identify covariates that explain the highest variation in the outcome variable. Model assumptions such as multicollinearity and influential values were tested to ascertain a good fit for the model, and a *p* value of < 0.05 was considered significant in the final model.

### 2.9. Ethics Review and Approval

The protocol was approved by the following ethics and research committees: the Kenyatta National Hospital-University of Nairobi Ethics and Research Committee (Approval No: P383/07/2020); the Biosafety, Ethics, and Animal Use Committee of the Faculty of Veterinary Medicine, University of Nairobi (Approval No: FVM BAUEC/2020/248); and the National Commission for Science Technology and Innovation (Approval No: NACOSTI/P/22/16,025).

## 3. Results

A total of 718 stool samples were collected from 361 individuals across different wards: 357 samples in ethanol and 361 in formalin. The distribution was as follows: Suswa (85/718, 12%), Melelo (72/718, 10%), Maji Moto-Naroosura (376/718, 52%), Mosiro (171/718, 24%), and Ololulung'a Ward (14/718, 2%). The majority of participants (98.6%) were above 18 years old. Gender representation was nearly equal, with 188 males (52%) and 173 females (48%).

### 3.1. *Taenia* spp. Detection Using Multiple Methods

From a total of 1078 analyses (see Table 1), a single stool sample from Mosiro Ward tested positive on the coproAg-ELISA (1/360 samples analyzed). All other samples tested negative on all tests. In total, stool samples from 353 individuals were subjected to all three tests. A summary of the findings is depicted in Table 1.

### 3.2. Detection of Other Intestinal Parasites

Copromicroscopy detected *Hymenolepis nana, G*. *lamblia, E*. *histolytica*/*dispar*/*moshkovskii, Chilomastix mesnili, Iodamoeba buetschlii*, and *Entamoeba coli.* The prevalence of infection with any pathogenic parasite detected on microscopy was 20.8%. The two most common pathogenic parasites detected were *E*. *histolytica*/*dispar*/*moshkovskii* (15.5%) and *G*. *lamblia* (5.3%), while the most abundant nonpathogenic protozoan detected was *E*. *coli* (10.5%). Helminths were not common in the samples analyzed. Polyparasitism of *E. histolytica*/*dispar*/*moshkovskii* and other intestinal parasites were observed. The point estimate of *E*. *histolytica*/*dispar*/*moshkovskii* prevalence in the different wards ranged between 9% and 29% while that of *G. lamblia* ranged from 0% to 19%. A summary of the findings is illustrated in Tables 1, 2, and 3.

### 3.3. Exploring Associations of Select Predictors and Infection With 2 Common Pathogenic Parasites

Putative risk factors associated with *E. histolytica*/*dispar*/*moshkovskii* and *G. lamblia* infections were explored. No significant findings were identified between *E. histolytica*/*dispar*/*moshkovskii* positivity in stool and the exposure variables (*p* value ≤ 0.05) at any stage of analysis. Factors identified to be associated with *G. lamblia* infection were formal education status and grazing distance from the homestead. Individuals who responded to have had formal education were 94% less likely to be infected by *G. lamblia*. Similarly, those who grazed their livestock near their homestead (approx. ≤ 2 km) were 93% less likely to test positive for *G. lamblia*. No statistically significant difference in *G. lamblia* infection was observed between genders. See Tables 4 and 5 for additional outputs of tests for association.

## 4. Discussion

The test prevalence of *Taenia* spp. was estimated at 0.3% and 0% using coproAg-ELISA and copromicroscopy, respectively. PCR assay did not detect *T*. *saginata* or *T. solium*. These findings align with earlier estimates reported by Wardrop et al. [17], Odongo et al. [18], Fèvre et al. [19], and Anyango et al. [49] who reported similar outcomes of 0%–0.3% occurrence in studies conducted in Kenya. However, some studies in the country have reported higher estimates ranging from 1.9% to 19.7% [12–16, 19], indicating variability in prevalence.

Several possibilities may explain the low estimates in this study. *Taenia saginata* sheds proglottids intermittently [6], and therefore, reliance on visual identification of parasites in stool may underestimate prevalence. This ultimately reduces the sensitivity of copromicroscopy coupled with the fact that only a small volume of stool is examined [50]. Serial sampling of the same individual may improve the chances of egg recovery, especially in a population where the prevalence is suspected to be low [51]; in this study however, sampling was conducted once. In addition, copromicroscopy may fail to detect infections when the cestode is immature. One stool sample (1/360) tested positive for *Taenia* spp. on coproAg-ELISA, and the same sample was negative on multiplex coproPCR and prior screening using microscopy. *Taenia* species coproAg-ELISA detects parasite antigens in stool, enabling detection before reproductive maturity of the cestode is attained and eggs are excreted [22, 24, 48]. In epidemiological settings, this assay has been lauded to detect 2.5 times more cases of taeniasis than microscopy [52]. It is, therefore, possible that the tapeworm carrier, in this case, harbored an immature taenia infection. Other surveillance studies that have simultaneously utilized microscopy, PCR, and coproAg-ELISA have seemingly reported relatively higher positivity rates in the latter [19, 24, 53]. However, the immunodiagnostic test does not differentiate between specific *Taenia* species [22, 52]. The study population was purposively constituted of pastoralist communities who do not practice pig farming and the consumption of pork [54]. Therefore, the probability of infection with *T*. *solium* was very low. The possibility of the positive test result stemming from cross-reactions from antigens from other parasite species cannot be ignored as the specificity of the test is not 100% [52]. *Taenia* species DNA was not detectable in the sample that was positive on coproAg-ELISA and this is contrary to findings by Yamasaki et al. [24] who were able to demonstrate DNA from nonegg sources in known carriers prior to patency. The PCR test has been reported to have a lower diagnostic sensitivity when compared to the coproAg-ELISA test [24].

The low *Taenia* spp. burden reported in this study suggests that there may be very few tapeworm carriers in the representative population who maintain the transmission cycle. This is a common epidemiological scenario [55–58]. The Narok region was previously described to have “hyperendemic pastoral cysticercosis,” characterized with a dual high burden of both *T. saginata* taeniasis and bovine cysticercosis [3, 9]. There has been a growing adoption of protective practices such as the use of latrines and hand washing over the years in the county albeit the water, sanitation, and hygiene capacity (WASH) in the county is not yet adequate [59]. These practices prevent infections [3, 49]. Notably, a related study conducted in the same county suggested the existence of spatial infection foci for bovine cysticercosis [60]. A targeted approach to surveillance, employing “absence of disease” methods [45], may be beneficial in further exploring the epidemiology of *T. saginata* infections. This approach could primarily focus on monitoring bovine cysticercosis in the cattle population. Complementary surveillance in human populations would enhance these efforts by identifying carriers.

Coprological examination was able to detect pathogenic intestinal protozoans such as *G. lamblia* and *Entamoeba* spp. Commensals such as *C*. *mesnili, I*. *buetschlii,* and *E*. *coli* were also identified. However, due to morphological similarities among *E. histolytica, Entamoeba dispar,* and *Entamoeba moshkovskii* [46], the true burden of the virulent *E. histolytica* could not be established. Previously published reports on parasitic intestinal protozoal infections in pastoral communities of the Narok region in Kenya are scarce. The prevalence estimates for *E*. *histolytica*/*dispar*/*moshkovskii* (15.5%) was similar to those reported by Emisiko et al. [34] and Mulinge et al. [40] (12%–18%, on microscopy). Higher estimates have been reported elsewhere [31].

The occurrence rate of *G. lamblia* reported (5.3%) were similar to those of previous studies by Chege et al. [33] and Njambi et al. [35] (4%–8%). Higher estimates have however been reported in the country, in studies majorly focusing on the infection burden in children living in informal settlements [31, 32, 34, 36, 37]. Assessment of inter-ward prevalence indicated that Suswa Ward had a higher point prevalence (19%) than the other four wards and a significantly higher burden of *G. lamblia* infection cases when compared to Maji Moto–Naroosura Ward (3%), and this information may be useful when planning intervention. These parasites (*Entamoeba* spp. and *G. lamblia*) share transmission pathways, that is, fecal–oral route, and their presence is suggestive of inadequate sanitation/hygiene and inaccessibility to potable water [61]. Narok County is characterized by open defecation practices, with only 35% having access to improved sanitation and 20% to improved drinking water sources [21, 43]. Open defecation leads to the contamination of pastures, soil, and surface water, increasing the risk of intestinal parasitic infections [35]. Additionally, the region is predominantly composed of pastoral communities. In Kenya, *G. lamblia* infections have been linked to contact with domestic animals, including cattle [38].

Grazing livestock near the homestead (approx. ≤ 2 km) was linked to a reduced likelihood of *G*. *lamblia* infection, suggesting that these individuals had better access to safe water, hygiene, and sanitation facilities, thereby decreasing the likelihood of fecal–oral transmission of the parasite. Pastoralism, which involves moving livestock to different grazing areas to meet their nutritional needs [54], may lead to reduced access to sanitation facilities. Access to water for sanitation has been reported to reduce intestinal protozoal infections [35]. Similarly, individuals with formal education were less likely to test positive for *G. lamblia* infection. This association may be attributed to food hygiene practices, which are often promoted through formal education, fostering awareness of basic hygiene principles and potentially influencing behavior [62, 63]. Health literacy additionally promotes health-seeking behavior and encourages practices such as regular prophylactic deworming [64]. This may also explain the low helminth burden in the sampled population.

Parasitic helminths such as hookworm and *H*. *nana* were also identified. The burden of hookworm in the sampled population was low. Similar prevalence estimates (< 1%) have been reported in the country by Steinbaum et al. [65], Sakari et al. [66], Steinbaum et al. [67], and Chege et al. [33]. Higher estimates have been reported by Nguhiu et al. [68, 69]. The low prevalence of STH in this study was anticipated as respondents consisted mainly of adults (98.6%). STH infections are common among pediatric populations [26]. Narok County has benefited from MDA initiatives targeting STH, primarily focused on school-going children [69]. Despite these efforts, the decline in the burden of STH in this demographic has been slow, leading to speculation that adults may act as reservoirs for these infections [43]. However, these findings suggest otherwise and support the current practice of targeting children in MDA programs. The prevalence of *H. nana* was similar to that estimated by Nguhiu et al. [68] and Mbae et al. [31] (≤ 1%). Sakari et al. [66] reported a slightly higher point estimate (3.6%). *Hymenolepis nana* infections are usually asymptomatic but have been linked to diarrhea in immunocompromised individuals [70]. Despite being of minor clinical significance, they serve as significant proxies indicating poor hygiene and sanitation practices, which may predispose individuals to more virulent intestinal pathogens due to shared transmission pathways. For instance, in this study, *H. nana* co-occurred with *E. histolytica*/*dispar*/*moshkovskii*. Humans and/or rodents are the definitive hosts for *H. nana*, and peridomestic rodents are known putative reservoirs and vectors for major zoonotic pathogens that underlie human disease [71].

Polyparasitism was observed in 5/361 of the stool samples, mainly involving *E*. *histolytica*/*dispar*/*moshkovskii* with one other enteric parasite. Similar observations have been made elsewhere [31–33, 68]. The health consequences of acute parasitic coinfections of the gut remain a largely underexplored area with conflicting findings [72]. The burden of pathogenic intestinal parasites in the sampled population was relatively high at 20.8% (copromicroscopy). These infections can all be traced to poor hygiene and sanitation practices, which, if addressed, offer an opportunity for substantial public health benefits. One Health–based solutions have been reported to be effective in such complex environments, as they integrate human, animal, and environmental health strategies to create sustainable and comprehensive interventions that save lives and safeguard livelihoods [73].

## 5. Conclusion

The estimated prevalence of human taeniasis appears to be very low in Narok County, Kenya. However, these individuals may be responsible for bovine cysticercosis hotspots, as reported in a related study conducted in the same area [60]. Collectively, these findings suggest that the Narok region no longer experiences hyperendemic *T. saginata* infections. While *G. lamblia* and *E. histolytica*/*dispar*/*moshkovskii* were the most prevalent pathogenic intestinal parasites, reports on these infections within the Narok region are scarce, despite the population's vulnerability to infection. The presence of these parasites indicates poor sanitation, insufficient meat inspection, and culinary habits that favor transmission.

An integrative approach to surveillance and control would yield significant public health gains in addressing the burden of the multiple pathogens identified. Strict adherence to meat inspection regulations, including the treatment or condemnation of carcasses infected with bovine cysticercosis and proper documentation of cases, would complement community-level interventions aimed at improving WASH capacity. Controlling *T. saginata* infections in humans would also require raising awareness about the importance of properly cooking beef and regular deworming. Strengthening WASH capacity would additionally alleviate the burden of pathogenic intestinal protozoal infections. Given the pastoral community context, a One Health approach to surveillance is crucial for investigating the roles of children, livestock, wildlife, and water bodies in the epidemiology of these infections. Integrative control initiatives could work synergistically to disrupt pathogen transmission pathways, safeguarding human, animal, and environmental health.

## Figures and Tables

**Table 1:** Prevalence of *Taenia* spp. detected by microscopy, coproantigen enzyme-linked immunosorbent assay, and multiplex polymerase chain reaction and estimates of other intestinal parasites identified through microscopy, Narok County, June–July 2022.

Parasite identified	Test					
	Microscopy (*n* = 361)		*Taenia* spp. mPCR (*n* = 357)		Coproantigen ELISA (*n* = 360)	
	No. positive results	Prevalence and 95% CI	No. positive results	Prevalence and 95% CI	No. positive results	Prevalence and 95% CI
*Taenia saginata*			0	0 and (0, 1.1)		
*Taenia solium*			0	0 and (0, 1.1)		
*Taenia* spp.	0	0 and (0, 1.1)			1	0.3 and (0, 1.6)
Hookworm (*Ancylostoma duodenale* or *Necator americanus*)	1	0.3 and (0, 1.6)				
*Hymenolepis nana*	4	1.1 and (0.4, 2.8)				
*Giardia lamblia*	19	5.3 and (3.4, 8.1)				
*Entamoeba histolytica*/*dispar*/*moshkovskii*	56	15.5 and (12.1, 19.6)				
*Any pathogenic parasite*	75	20.8 and (16.9, 25.3)				
*Chilomastix mesnili *⁣^∗^	4	1.1 and (0.4, 2.8)				
*Iodamoeba buetschlii *⁣^∗^	27	7.5 and (5.2, 10.7)				
*Entamoeba coli *⁣^∗^	38	10.5 and (7.8, 14.1)				

⁣^∗^Nonpathogenic.

**Table 2:** Ward-level prevalence estimates of *Entamoeba histolytica*/*dispar*/*moshkovskii* and *Giardia lamblia,* Narok County, June–July 2022.

Ward	Stool samples retrieved	*Entamoeba histolytica*/*dispar*/*moshkovskii* prevalence and 95% CI	*G. lamblia* prevalence and 95% CI
Suswa	43	9 (3, 22)	19 (8, 33)
Melelo	36	11 (3, 26)	3 (0, 15)
Maji Moto–Naroosura	190	14 (10, 20)	3 (1, 7)
Mosiro	85	22 (14, 33)	5 (1, 12)
Ololulung'a	7	29 (4, 71)	0 (0, 41)

**Table 3:** Polyparasitism identified on stool analysis by microscopy, Narok County, June–July 2022.

Coinfections	Microscopy (*n* = 361)	
Parasites identified	No. positive	Prevalence and 95% CI
*Entamoeba histolytica*/*dispar*/*moshkovskii + Giardia lamblia*	3	0.8 and (0.3, 2.4)
*Entamoeba histolytica*/*dispar*/*moshkovskii + Hymenolepis nana*	1	0.3 and (0, 1.6)
*Entamoeba histolytica*/*dispar*/*moshkovskii + Hookworm*	1	0.3 and (0, 1.6)

**Table 4:** Characteristics of study respondents with *Giardia lamblia* infection, Narok County, June–July 2022.

Respondent characteristics		Overall, *n* = 361	No. negative, *n* = 342	No. positive, *n* = 19	*p* value
Position in household	Dependent	90 (25%)	87 (25%)	3 (16%)	0.4
	Guardian	271 (75%)	255 (75%)	16 (84%)	

Sex	Female	173 (48%)	165 (48%)	8 (42%)	0.6
	Male	188 (52%)	177 (52%)	11 (58%)	

Education	None	147 (41%)	142 (42%)	5 (26%)	0.2
	Formal	214 (59%)	200 (58%)	14 (74%)	

Grazing distance	Far (> 2 km)	201 (56%)	183 (54%)	18 (95%)	< 0.001⁣^∗^
	Near	160 (44%)	159 (46%)	1 (5.3%)	

Grazing system	Private	128 (35%)	123 (36%)	5 (26%)	0.4
	Communal	233 (65%)	219 (64%)	14 (74%)	

Water source for livestock	Rivers	175 (48%)	168 (49%)	7 (37%)	0.3
	Waterpans	186 (52%)	174 (51%)	12 (63%)	

Water source for bathing	Not shared	88 (24%)	85 (25%)	3 (16%)	0.6
	Shared	273 (76%)	257 (75%)	16 (84%)	

Distance of water source from homestead	Far	107 (30%)	101 (30%)	6 (32%)	0.9
	Near	252 (70%)	239 (70%)	13 (68%)	
	Unknown	2	2	0	

Latrines	Absent	193 (53%)	188 (55%)	5 (26%)	0.015⁣^∗^
	Present	168 (47%)	154 (45%)	14 (74%)	

Gut symptoms	Absent	88 (24%)	82 (24%)	6 (32%)	0.4
	Present	273 (76%)	260 (76%)	13 (68%)	

Meat preparation methods	Boiling	339 (94%)	321 (94%)	18 (95%)	> 0.9
	Roasting	22 (6.1%)	21 (6.1%)	1 (5.3%)	

Age in years	12–23	49 (14%)	47 (14%)	2 (11%)	0.3
	24–45	285 (79%)	271 (79%)	14 (74%)	
	46 >	27 (7.5%)	24 (7.0%)	3 (16%)	

⁣^∗^*p* value of < 0.05.

**Table 5:** Associations between respondent characteristics significant in the final model and *Giardia lamblia* infection.

Characteristic	Crude odds ratio			Adjusted odds ratio		
	OR	95% CI	*p* value	OR	95% CI	*p* value
Education						
None	—	—	—	—	—	—
Formal	1.99	0.74, 6.26	0.2	0.06	0.01, 0.50	0.014⁣^∗^
Grazing distance						
Far (> 2 km.)	—	—	—	—	—	—
Near	0.06	0, 0.31	0.008⁣^∗^	0.07	0.00, 0.36	0.011⁣^∗^
Latrines						
Absent	—	—	—	—	—	—
Present	1.23	0.74, 2.05	0.4	3.49	0.97, 14.5	0.067

⁣^∗^*p* value of < 0.05.

## Data Availability

The data that support the findings of this study are available from the corresponding author upon reasonable request.
